# *COMT-Val158Met* polymorphism modulates antipsychotic effects on auditory verbal hallucinations and temporal lobe gray matter volumes in healthy individuals—symptom relief accompanied by worrisome volume reductions

**DOI:** 10.1007/s11682-019-00043-5

**Published:** 2019-02-02

**Authors:** Chuanjun Zhuo, Langlang Cheng, Gongying Li, Yong Xu, Rixing Jing, Shen Li, Li Zhang, Xiaodong Lin, Chunhua Zhou

**Affiliations:** 1 Department of Psychiatric-Neuroimaging-Genetics and Morbidity Laboratory (PNGC-Lab), Tianjin Mental Health Center, Mental Health Teaching Hospital, Nankai University Affiliated Anding Hospital, Tianjin Medical University, Tianjin, 300222 China; 2 Department of Psychiatry, Institute of Mental Healthy, Genetic Lab, Jining Medical University, Jining, 272191 China; 3 Department of Psychiatry, College of Basic Medical Research, Tianjin Medical University, Tianjin, 300000 China; 4 Department of Psychiatry, First Hospital/First Clinical Medical College of Shanxi Medical University, Taiyuan, 030001 China; 5 Department of Psychiatry, Wenzhou Seventh People’s Hospital, Wenzhou, 325000 China; 6 Department of Psychiatry, Shanxi Medical University, Taiyuan, 030000 China; 7 Department of Pattern Recognition, China National Key Laboratory, Institute of Automation, Chinese Academy of Sciences, Beijing, 100191 China; 8 Department of Pattern Recognition, University of Chinese Academy of Sciences, Beijing, 100191 China; 9 GHM Institute of CNS Regeneration, Jinan University, Guangzhou, 510632 Guangdong Province China; 10 Department of Pharmacy, The First Hospital of Hebei Medical University, Shijiazhuang, 050000 China

**Keywords:** Auditory verbal hallucination, Anti-psychotic, Hi-AVHs, COMT, MRI

## Abstract

Investigation of auditory verbal hallucinations (AVHs) in schizophrenics is complicated by psychiatric symptoms. Investigating healthy individuals with AVHs (H-AVHs) can obviate such confounding factors. The objective of this study was to explore the effects of antipsychotic treatment on AVHs and gray matter volumes (GMVs) in H-AVH subjects and whether such are effects are influenced by *COMT*-*Val158Met* genotype. Magnetic resonance imaging (MRI) and genotyping studies were completed for 42 H-AVH subjects and 42 well-matched healthy controls (HCs). *COMT-Met/Met* homozygotes (158th codon) were identified as COMT-Met genotype; *COMT-Met/Val* heterozygotes and *COMT-Val/Val* homozygotes were identified as COMT-Val genotype. Data were compared across groups (H-AVH vs. HC, and between genotypes) with two-sample t-tests. The H-AVH COMT-Met group showed a stronger response to antipsychotic treatment than the H-AVH COMT-Val group (*p* < 0.001). Both H-AVH genotype groups exhibited temporal lobe GMV reductions after treatment, and relative to their respective genotype-matched HC groups. Antipsychotic treatment effects in H-AVH subjects were influenced by *COMT-Val158Met* genotype and associated with widespread GMV reductions. These findings provide clues for further exploration of treatment targets for AVHs. Treatment associated GMV reductions, however, raise concerns about use of antipsychotics in H-AVH subjects.

## Introduction

Emprirical studies have indicated that some 0.7% of the general population has experienced auditory verbal hallucinations (AVHs) defined based on Jhons’ strict criteria (Johns et al. 2004; Sommer et al. 2010; Upthegrove et al. 2016). Various hypotheses have been proposed to explain the AVHs in patients with schizophrenia from different perspectives (Alderson-Day and Fernyhough 2016; Alderson-Day et al. 2017; Alderson-Day et al. 2015; Baumeister et al. 2017; Cho and Wu 2014; Conde et al. 2016; Curcic-Blake et al. 2017; Hugdahl 2015; Jones 2010; Liemburg et al. 2012; McCarthy-Jones et al. 2014; Northoff 2014; Wilkinson 2014; Wilkinson and Fernyhough 2017); however, none of the proposed hypotheses has achieved general acceptance (Wilkinson and Fernyhough 2017). To the best of our knowledge, no published research has focused on investigating the effects of antipsychotic medication on AVHs and accompanied brain alterations in H-AVH subjects.

The efficacy of antipsychotic drugs in patients with schizophrenia has been shown to be related to catechol-o-methyl transferase (COMT) genotype at the 158th codon, where a valine-to-methionine substitution (rs4680) is common (Huang et al. 2016; Olgiati et al. 2009; Sagud et al. 2010). COMT with *Val* in this location is much more efficient at removing dopamine than COMT with *Met* at this location. Hence, COMT-*Val/Val* homozygotes exhibit very efficient COMT activity, COMT-*Met/Met* homozygotes exhibit hypo-efficient COMT activity, and *Val/Met* heterozygotes carrying both variants of the enzyme exhibit an intermediate activity level, generally within normal range. *COMT-Val158Met* genotype (COMT genotype from here forward for simplicity) has also been shown to influence structural and functional aspects of auditory processing, including dopaminergic alterations in both healthy subjects and patients with schizophrenia (Edgar et al. 2012; Gothelf et al. 2011; Kang et al. 2010; Li et al. 2015; Lu et al. 2007; Steiner et al. 2018; Tian et al. 2013a; Tian et al. 2013b). Hence, a convergence of findings indicates that there may be reciprocal interactions between COMT genotype, dopamine levels, and structural/functional brain alterations in relation to neuropsychiatric symptoms, such as AVHs (Edgar et al. 2012; Gothelf et al. 2011; Huang et al. 2016; Kang et al. 2010; Li et al. 2015; Lu et al. 2007; Sagud et al. 2010; Steiner et al. 2018; Tian et al. 2013a; Tian et al. 2013b).

Schizophrenics with the *COMT*-*Met*/*Met* genotype respond more strongly to antipsychotics, in terms of positive symptom alleviation, than schizophrenics with the *COMT*-*Val/Val* genotype, and this response is associated with characteristic brain structural alterations (Edgar et al. 2012; Gong et al. 2016; Lei et al. 2015). Although AVHs are a classic positive symptom of schizophrenia (Reed et al. 2018; Tandon 2013), no study has reported the effects of antipsychotics on AVHs per se in schizophrenic patients. Typically, AVHs have been encompassed within a positive symptom cluster without explicit distinction (Huang et al. 2016; Olgiati et al. 2009; Sagud et al. 2010). To the best of our knowledge, there has been one study that has reported that adjuvant transcranial direct current stimulation alleviated AVHs more effectively in schizophrenia patients with a *COMT-Met/Met* genotype than in those with a *COMT-Val/Val* genotype (Chhabra et al. 2018). A recent systematic review reported that antipsychotics can improve AVHs in patients with borderline personality disorder (Slotema et al. 2018), which suggests that it may be feasible to explore antipsychotic effects on isolated AVHs. Moreover, several studies have recommended possible antipsychotic use to treat AVHs in otherwise healthy patients (Snitz et al. 2006; de Leede-Smith and Barkus 2013; Upthegrove et al. 2016; Vallath et al. 2018).

Exploratory studies of H-AVH subjects, including examining the effects of antipsychotic treatment, can provide fundamental information about the mechanisms underlying AVHs. Investigation of AVHs in H-AVH subjects can provide important information to help clarify the precise pathological features of AVHs and avoid many confounding factors, such as other psychiatric symptoms (Hugdahl 2015; Jones 2010; Wilkinson and Fernyhough 2017).

It is not yet clear how COMT genotype may be related to AVH severity in H-AVH subjects, particularly with respect to brain structural alterations and the effectiveness of antipsychotics for treating AVHs. We hypothesize that the effectiveness of antipsychotic treatment for AVH symptoms in H-AVH subjects may be influenced by COMT genotype and that this genotype variation will have accompanying structural brain alterations. In the present study, we employed genotyping and magnetic resonance imaging (MRI) with statistical parametric mapping (SPM) techniques to explore the influence of COMT genotype on AVH symptoms and the effectiveness of a 6-month antipsychotic drug treatment regimen on AVHs and gray matter volumes (GMVs) in H-AVH subjects.

## Materials and methods

### Sample

We used advertisements in 1000 local communities (total resident population > 200,000) to recruit H-AVH volunteers from January 1, 2016 to June 31, 2018. We enrolled 300 healthy people with diagnosed AVHs; among them, 115 subjects reported that they had suffered mental distress caused from the AVHs and volunteered to accept pharmacological treatment with risperidone (Johns and Johns, Xi’an Yang-Sen Pharmaceutical Co., Ltd.) at dosages in the range of 100–500 mg/d (chlorpromazine equivalent dosing). Exclusion criteria included diagnosis of any other mental disorder by psychiatrist, according to the Structured Clinical Interview for DSM-5, or diagnosis of a neurological disease by a neurologist according to standard neurological diagnostic criteria. From a cohort of subjects who participated in a prior pilot study, we recruited healthy control (HC) subjects matched to the H-AVH subjects with respect to COMT genotype, gender, age, and education level. The Tianjin Anding Hospital ethics review board approved this study and all patients provided written consent. The assessments were carried out in compliance with the Declaration of Helsinki guidelines and approved by the institutional ethics committee.

### Self-report assessments

We used the Wisconsin Card Sorting Test (WCST, Westwood et al. 2016) and global assessment scale (GAS, Dauwan et al. 2016) to monitor benefit/risk ratios in H-AVH subjects. AVHs were assessed with the auditory verbal hallucinations rating scale (AHRS) (Haddock et al. 1999). The AHRS, which is one of two components of the Psychotic Symptom Rating Scales instrument set (the other assesses delusions), was shown to have excellent inter-rater reliability. The AHRS consists of 11 items addressing the following aspects of hallucinations: negative content amount; negative content degree; distress amount; distress intensity; frequency; duration; loudness; disruption; control; location; and (beliefs regarding) origin distress.

### Genotyping

Blood collection and genotyping were performed as previously reported (Chhabra et al. 2018). Briefly, 5 ml of peripheral blood was collected in K_2_EDTA-treated vacutainers (Becton & Dickinson, Franklin Lakes, NJ), and genomic DNA was extracted using commercial spin columns (Qiagen, Inc., Limburg, the Netherlands). The quality of extracted DNA was determined by ultraviolet spectrophotometry (Thermo Scientific, Waltham, MA). We submitted genomic DNA subjected to COMT genotyping at rs4680 using the TaqMan 5′ nuclease allelic discrimination assay. The genotyping was performed by real-time polymerase chain reaction (PCR) in a 96-well plate (StepOne Plus™ Real-Time PCR Systems, Applied Biosystems) with predesigned, commercially available primers and allele-specific minor groove binding probes (FAM and VIC; Applied Biosystems, Foster City, CA) in a reaction volume of 10 μl (10 ng of genomic sample DNA, assay mix and PCR Universal Master Mix with AmpErase® uracil-DNA glycosylase) as follows: 60 °C for 30 s, and 95 °C for 10 min, followed by 50 cycles of 92 °C for 15 s and 60 °C for 90 s. PCR was performed in duplicate with both positive and negative controls. Genotypes were grouped by allele dominance (Chhabra et al. 2018). That is, *COMT-Met/Met* homozygotes were regarded as having the COMT-Met genotype and *COMT-Met/Val* heterozygotes and *COMT-Val/Val* homozygotes were regarded as having the COMT-Val genotype (Kang et al. 2010).

### Imaging data acquisition

All MRI data were obtained on a 3.0-T MR system (Discovery MR750, General Electric, Milwaukee, WI). Tight but comfortable foam padding was used to stabilize head position, and earplugs were used to reduce scanner noise during image acquisition. A three-dimensional T1-weighted brain volume sequence with 188 sagittal slices was performed with the following parameters: repetition time = 8.2 ms; echo time = 3.2 ms; inversion time = 450 ms; flip angle = 12°; field of view = 256 mm^2^; matrix = 256 × 256; slice thickness = 1 mm, no gap.

### GMV calculation

Voxel-wise GMVs were calculated by SPM in SPM8 software (http://www.fil.ion.ucl.ac.uk/spm/software/spm8/). Employing the standard unified segmentation model, we segmented images into gray matter, white matter, and cerebrospinal fluid. After affine registration of the gray matter concentration map into Montreal Neurological Institute space with diffeomorphic anatomical registration and exponentiated Lie algebra (DARTEL), gray matter concentration images were warped nonlinearly and converted to a 1.5-mm^3^ voxel size. The nonlinear determinants were derived from the spatial normalization step and multiplied by the gray matter concentration map to obtain the GMV of each voxel. GMV images were smoothed with a 6-mm^3^ full width at half maximum Gaussian kernel. The normalized, modulated, and smoothed GMV maps were used for statistical analyses after spatial preprocessing as described in detail previously (Zhuo et al. 2017).

### Statistical analysis

Means are reported with standard deviations (SDs). A two-sample t-test was used to compare GMVs between groups (H-AVH vs. HC, and COMT-Met vs. COMT-Val) and time points (baseline vs. after 6 months of antipsychotic treatment) in a voxel-wise manner with adjustment for age and sex. The family-wise error method was used to correct for multiple comparisons (*p* < 0.05).

## Results

### Group characteristics

Ultimately, 34 COMT-Met and 45 COMT-Val H-AVH subjects underwent dopamine antagonist treatment for 6 months. We obtained complete and fully analyzable MRI data from 25 COMT-Met subjects and 21 COM-Val subjects (at baseline and 6 months later). We factitiously discarded data from 3 COMT-Met subjects and 1 COMT-Val subject (see limitations paragraph in the Discussion), preserving 22 COMT-Met and 20 COMT-Val H-AVH subjects for further analysis. The two H-AVH genotype groups did not differ significantly with respect to gender ratio, age, educational level, AVH duration, and AVH symptom severity. The sociodemographic, genotype, and treatment response characteristics of the two H-AVH genotype groups are compared in Table 1. The HC group consisted of 22 COMT-Met and 20 COMT-Val subjects as well; the characteristics of the two HC genotype groups are summarized in Table 2.

**Table 1:** Sociodemographic and clinical characteristics of the Hi-AVHs genotype groups

Characteristic	COMT-Met	COMT-Val	*t/x*^*2*^	*p*
Mean age, years	24.91 ± 3.58	25.23 ± 3.61	−0.293	0.771
Gender, males:females	7:15	10:10	1.437	0.231
Mean education level	11.91 ± 2.33	11.86 ± 1.81	0.072	0.943
Mean AVH duration, months	128.73 ± 40.84	129.86 ± 43.69	−0.089	0.929
Mean risperidone dosage, mg/d, chlorpromazine equivalent	250.40 ± 50.10	300.15 ± 75.50	−2.564	0.014
Mean AHRS scores				
Baseline (Bl)	28.41 ± 4.25	28.32 ± 5.40	−0.279	0.781
Posttreatment (Pt)	12.18 ± 4.08	23.14 ± 5.89	6.781	0.000
Baseline vs. posttreatment	*t* = 8.341	*t* = 2.974	–	–
	*p* < 0.001	*p* = 0.005		

The auditory verbal hallucinations scale (AHRS, Gillian Haddock, University of Manchester, 1999) was used to assess AVH symptom severity

**Table 2:** Sociodemographic characteristics of the HC genotype groups

Characteristic	COMT-Met	COMT-Val	*t/x*^*2*^	*p*
Mean age, years	24.50 ± 2.50	25.00 ± 4.2	0.474	0.638
Gender, males:females	7:15	10:10	1.437	0.231
Mean education level, years	12.50 ± 3.68	13.50 ± 2.50	1.020	0.314

Antipsychotic dosage differed significantly between the two H-AVH genotype groups, with COMT-Val subjects receiving significantly higher risperidone dosages than COMT-Met subjects (Table 1). Despite their being treated with lower antipsychotic dosing, and otherwise comparable medication regimens, the treatment was markedly more effective at alleviating AVHs in COMT-Met H-AVH subjects than in COMT-Val H-AVH subjects (Table 1).

### Baseline GMVs

**Fig. 1:**
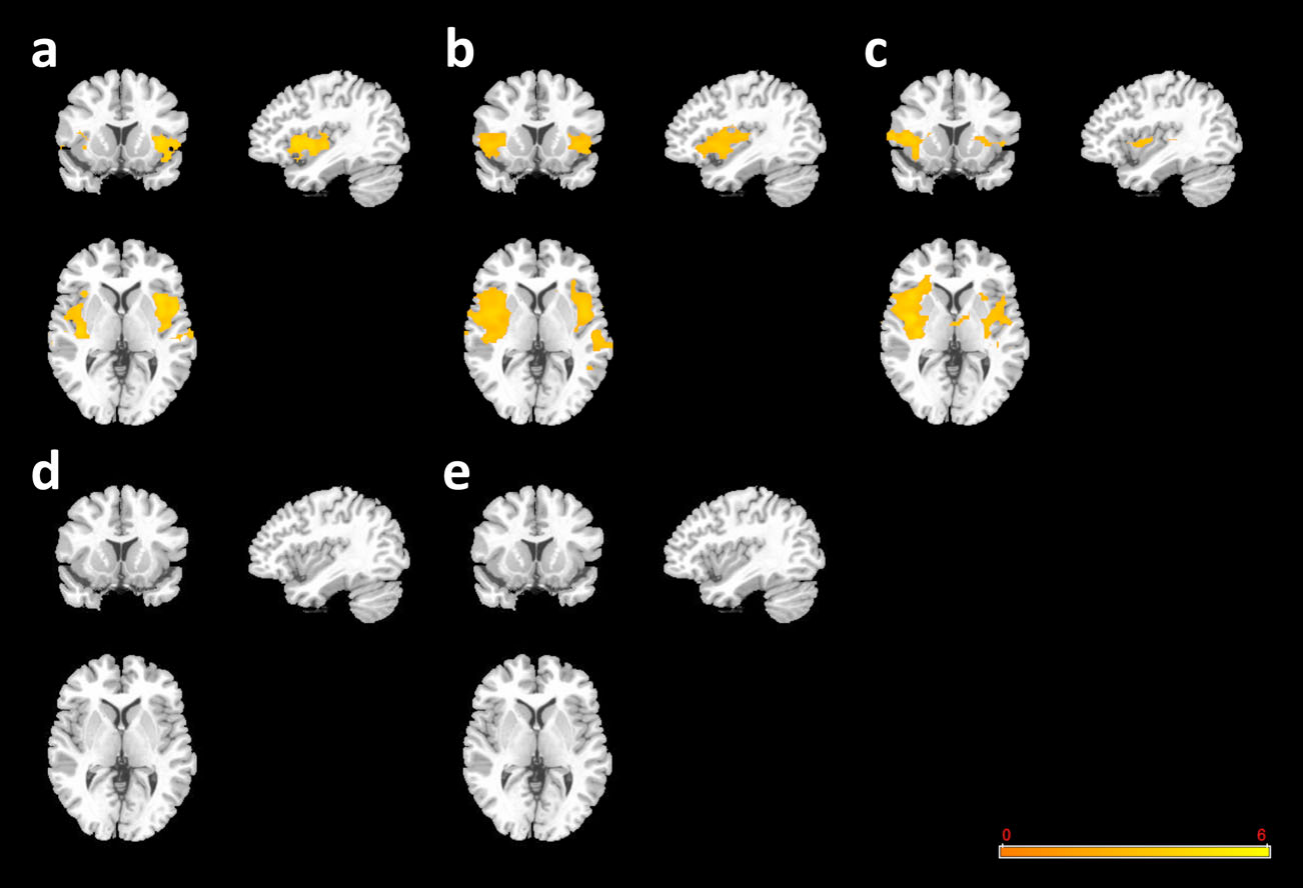
**GMV baseline comparisons. a** H-AVHs at baseline vs. HCs (genotype groups combined). **b** H-AVH COMP-Met group at baseline vs. HC COMT-Met group. **c** H-AVH COMP-Val group at baseline vs. HC COMT-Val group. **d** HC COMT-Met group vs. HC COMT-Val group. **e** H-AVH COMT-Met group at baseline vs. H-AVH COMT-Val group at baseline. Warm pseudocolor represents increased GMV in the former group relative to the second (reference) group

We observed enlarged GMVs, mainly in the temporal lobes, in all Hi-AVH subjects at baseline compared to GMVs observed in the HC reference group (Fig. 1a). Similarly, comparing GMVs in H-AVH subjects with the COMT-Met genotype versus GMVs in HCs with the same genotype, we found larger GMVs mainly in the temporal lobes (Fig. 1b). Interestingly, the scope of GMV differences between H-AVH subjects and HCs was more pronounced when only COMT-Met genotype groups were compared. H-AVH subjects with a COMT-Val genotype also had larger GMVs in their temporal lobes than HCs with a COMT-Val genotype (Fig. 1c). The GMV enlargements in the H-AVH COMT-Val group, however, were smaller, particularly in the left temporal lobe, than the enlargements seen for the whole H-AVH cohort and for the H-AVH COMT-Met genotype group. We did not observe significant GMV differences related to COMT genotype (Met vs. Val) within the HC (Fig. 1d) or H-AVH cohorts (Fig. 1e).

### Treatment effects on GMVs in H-AVH subjects

**Fig. 2:**
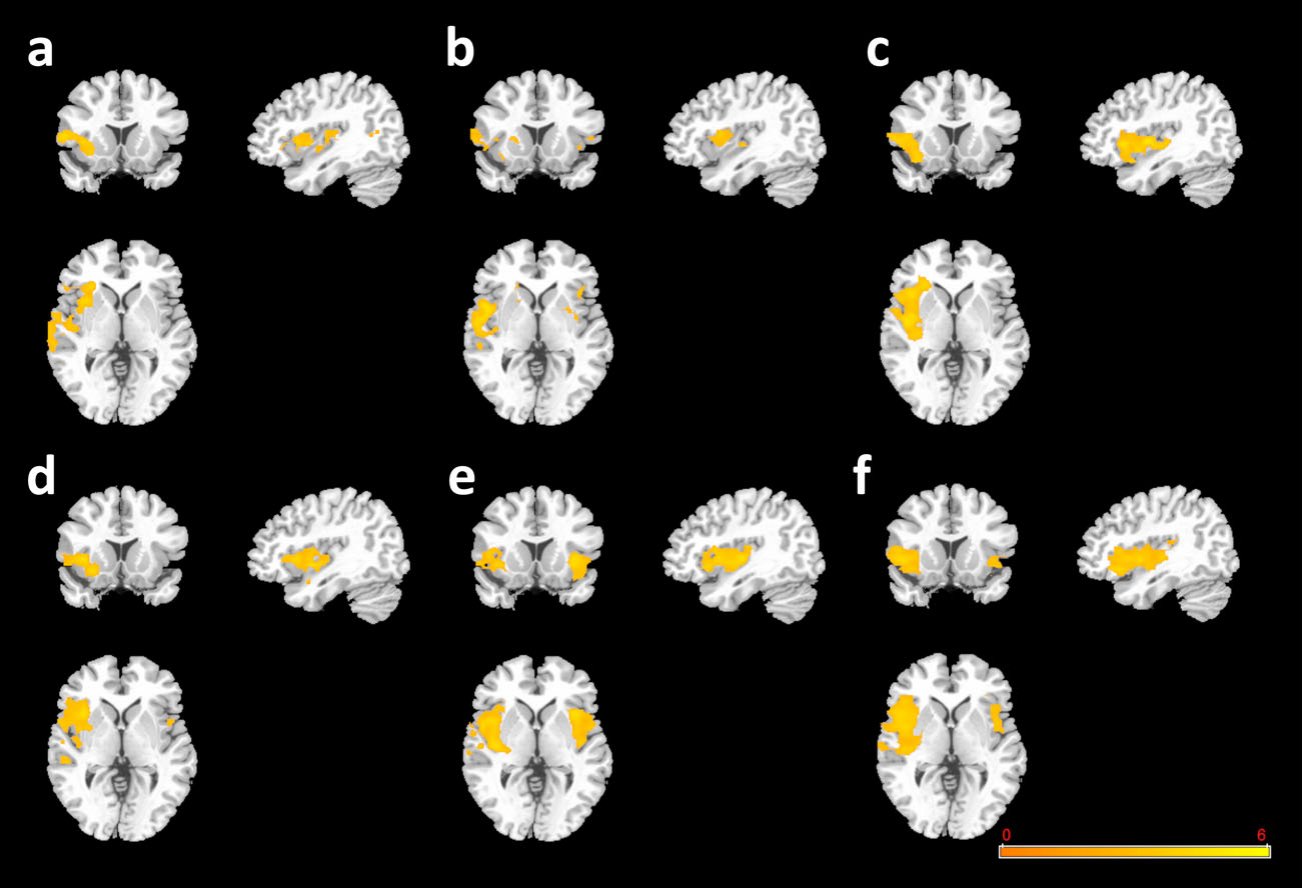
**Antipsychotic treatment effects on GMVs in H-AVH subjects**. **a** Posttreatment H-AVHs vs. HCs (both genotype groups combined). **b** Posttreatment H-AVH vs. baseline H-AVH (both genotype groups combined). **c** H-AVH COMT-Met group only at 6 months posttreatment vs. baseline. **d** H-AVH COMT-Val group only at 6 months posttreatment vs. baseline. **e** H-AVH COMT-Met group posttreatment vs. HC COMT-Met group. **f** H-AVH COMT-Val group posttreatment vs. HC COMT-Val group. Cool pseudocolor represents decreased GMV in the former group relative to the second (reference) group

After 6 months of risperidone treatment, we observed obvious GMV reductions in the H-AVH participants compared to HCs (Fig. 2a) and compared to their own pretreatment baseline scans (Fig. 2b), with the latter difference appearing to be more widespread. Looking only at H-AVH subjects with a COMT-Met genotype after versus before treatment, the GMV reduction pattern was more prominent than in the combined genotypes comparison, particularly in the right temporal lobe (Fig. 2c). Conversely, looking only at H-AVH subjects with a COMT-Val genotype after versus before treatment, the GMV reductions were less pronounced than in the H-AVH COMT-Met group (Fig. 2d). After 6 months of risperidone treatment, both H-AVH subjects with a COMT-Met genotype (Fig. 2e) and H-AVH subjects with a COMT-Val genotype (Fig. 2f) showed reduced GMVs in their temporal lobes relative to their genotype-matched HC groups.

### Cognitive status

WCST and GAS scores obtained for all H-AVH subjects were within normal range at baseline (before treatment) and remained within normal range after 6 months of risperidone treatment.

## Discussion

In the present study, we demonstrated for the first time that antipsychotic drug effects in H-AVH subjects are influenced by COMT genotype and that this distinction is accompanied by corresponding structural changes in the brain, particularly in the temporal lobes. Importantly, we compared GMVs after 6 months of risperidone treatment to pretreatment baseline GMVs within each H-AVH genotype group, providing supplementary information regarding the pathological features of H-AVH participants with specific COMT genotypes. Notably, alleviation of AVH symptoms was more pronounced in H-AVH subjects with a COMT-Met genotype than in H-AVH subjects with a COMT-Val genotype. Despite the limitations of this study (delineated below), our results provide clues to guide future studies.

Our findings of enlarged temporal lobe GMVs in H-AVH subjects, regardless of COMT genotype, compared to HCs, indicate that these alterations may reflect pathological features of AVHs, consistent with the hypotheses that AVHs may be related to structural abnormalities of the temporal lobe and that temporal lobe hyperactivity may be an intrinsic feature of AVH symptomology (Curcic-Blake et al. 2017; Hugdahl 2015; Kompus et al. 2011; Morch-Johnsen et al. 2017; Steinmann et al. 2014; Upthegrove et al. 2016; van Lutterveld et al. 2014; Wigand et al. 2015). Our findings of antipsychotic-induced reductions in temporal lobe GMVs provide additional indirect support for the hypothesis that an enlarged temporal lobe is an intrinsic feature of AVH symptomology (Hugdahl 2015; Kompus et al. 2011; Morch-Johnsen et al. 2017; Steinmann et al. 2014; van Lutterveld et al. 2014; Wigand et al. 2015). Our negative findings of no GMV differences between COMT-Met and COMT-Val genotype H-AVH groups at baseline suggest that, developmentally, COMT genotype does not influence temporal lobe GMV enlargement in H-AVH subjects, despite a genotype effect on AVH symptom severity and antipsychotic drug effectiveness for AVH symptom alleviation.

Partial correlation analysis and Pearson correlation analysis (according to variable properties) did not reveal any correlations among GMV alterations, AVH alterations, risperidone dosage, and duration of AVH symptoms at any examined time point (baseline, 6 months after treatment). These negative findings support the notion that AVH symptoms and antipsychotic-induced GMV reductions in H-AVH subjects may be related substantially to COMT genotype.

More importantly, we found that antipsychotic medication induced worrisome GMV reductions. Although many studies reported that antipsychotics can induce frontal-temporal GMV reduction in patients with schizophrenia (Ho et al. 2011; Andreasen et al. 2013; Lawrie 2018), the GMV reductions observed in our H-AVH participants in this study exceeded our expectations with respect to both scope and rapidity. These findings raise the concern that long-term antipsychotic use has the potential risk for widespread, detrimental GMV reductions. Therefore, we would not recommend antipsychotics as a first-line treatment for AVHs in otherwise healthy patients. Such patients may be better served by other approaches, such as psychotherapy, transcranial direct current stimulation, or avatar therapy (Dollfus et al. 2018; du Sert et al. 2018; Plewnia et al. 2018; Stephanie et al. 2018; Thomas et al. 2018; Craig et al. 2018).

### Limitations

This study has at least nine notable limitations, and we hope sincerely that constructive dialogue with international scholars will provide guidance for our subsequent studies. First, despite our multiple retention efforts, approximately half of the subjects did not complete the full 6-month study. Given the importance of longitudinal monitoring to clarify the dynamic trajectory of AVH characteristics, even greater efforts are needed to retain a large study sample. Second, to explore potentially objective evaluation indices, we discarded some subjects’ data due to excessive deviation from the bulk of the cohort (outlier exclusion). Moving forward from the present pilot study, it will be important to strengthen our methods to enable heterogeneous subject samples to be analyzed. Third, we adopted a relatively simple GMV metric for exploring brain alterations. We intend to apply more precise image data analysis methods in future studies. Fourth, we focused on COMT genotype; other genes (e.g. *FOX2* and *NRG1*) would be of interest to examine in a similar context. Furthermore, genomic, transcriptomic, and even proteomic methods may provide complementary information in the future. Fifth, we used a 3.0-T scanner though there are higher-resolution scanners (e.g. 7.0-T) in use in China. We hope that a strategic collaboration may enable us to conduct future studies with a higher resolution MRI scanner. Sixth, in this pilot study, we did not analyze different treatment periods or reciprocal gene interaction effects. Seventh, we did not include a schizophrenia patient comparison group. Eighth, we did not compare data between the participants who fully completed the study and those who did not. Thus, it is possible that those who did not complete the study did experience the same magnitude of symptom benefit and/or GMV reduction as the analyzed participants. Finally, we administered only the WSCT and GAS to monitor cognitive ability. We intend to administer more precise cognitive tests in future studies.

## Conclusion

COMT genotype was found to influence antipsychotic drug effects on AVH symptoms in H-AVH subjects. Compared to COMT-Val subjects, COMT-Met subjects responded more strongly to antipsychotic treatment with respect to both AVH symptoms and the magnitude of GMV reductions observed. Although the H-AVH subjects retained normal-range cognitive ability, as evidenced by the WSCT and GAS, throughout the study, the marked GMV reductions observed raise concerns that antipsychotic pharmacotherapy may not be well suited for H-AVH subjects. We would suggest that other approaches, such as psychological therapy, transcranial direct current stimulation, or avatar therapy (Dollfus et al. 2018; du Sert et al. 2018; Plewnia et al. 2018), be administered first to this otherwise healthy population. Despite the aforementioned limitations, these findings provide primary information toward explaining the mechanisms of AVHs and highlighting potential targets for AVH treatment in H-AVH subjects as well as, perhaps, schizophrenic patients.
